# EVERYDAY ACTIVITIES AND DEPRESSIVE SYMPTOMS: DOES GENDER MATTER?

**DOI:** 10.1093/geroni/igz038.3389

**Published:** 2019-11-08

**Authors:** Celeste Beaulieu, Jeffrey E Stokes

**Affiliations:** University of Massachusetts Boston, Boston, Massachusetts, United States

## Abstract

Previous research has suggested that informal socializing can be beneficial for mental health, whereas prior findings concerning solitary activities and mental health have been equivocal. Activity theory posits that involvement in activities – particularly social activities – can improve adults’ self-concept and self-esteem, leading to improved well-being. Solitary activities may perform the same function, though without any social reinforcement. However, social engagement and mental health may both vary by gender. Thus, we examined associations of informal socializing and solitary activities with depressive symptoms among 13,387 respondents of the 2012/2014 waves of the Health and Retirement Study, and further assessed potential gender differences. Results revealed that both informal socializing and solitary activities were significantly associated with lower depressive symptoms when analyzed separately. However, when both types of activities were modeled simultaneously, only informal socializing remained significant. Further, stratified analyses revealed that informal socializing was a significant predictor of depressive symptoms among women but not men, although these coefficients were not significantly different from each other. Overall, findings suggest that both informal socializing and solitary activities may be beneficial for mental health, yet results were clearly stronger for informal socializing. Socializing may benefit mental health not only by bolstering one’s self-concept, but also by linking adults with social ties and support networks that are instrumental for well-being in mid- and later life. Moreover, gender differences in effects were minimal and largely non-significant, indicating that activity involvement can bolster mental health for men and women alike.

